# How social innovations spread globally through the process of reverse innovation: a case-study from the South Korea

**DOI:** 10.1007/s43039-021-00027-8

**Published:** 2021-06-05

**Authors:** Chiara Cannavale, Lorenza Claudio, Michele Simoni

**Affiliations:** Department of Management and Quantitative Studies, Università degli Studi di Napoli Parthenope, Via Generale Parisi, 13, 80133 Napoli, NA Italy

**Keywords:** Social innovation, Reverse innovation, Case-study, South Korea

## Abstract

Nowadays, innovation is no longer a peculiarity of developed economies. Indeed, more frequently, it occurs that innovations born in the so called "emerging countries" spread in the advanced ones. This phenomenon is well known as Reverse innovation (RI), and within the global innovation literature about RI, some authors refer to these reversed innovations as developed in order to solve social or economic issues, specific of emerging contexts. However, scholars use to connect innovation with social goal as primary benefit to another phenomenon: i.e., Social innovation (SI). Within the Social innovation literature, there is a lack concerning how it should be undertaken to spread globally. Thus, we applied the Reverse innovation process to Social innovations: through a case-study analysis, we link the two phenomena which have never been explored together in previous studies. The paper aims at understanding how Social innovations spread from emerging to more advanced markets, while implementing this inversion of the flow. Further, we want to explore which is the potential that a Social innovation has in the host market: in other words, if SI could lose, hold, reduce, or increase their original social connotation.

## Introduction

**Fig. 1:**
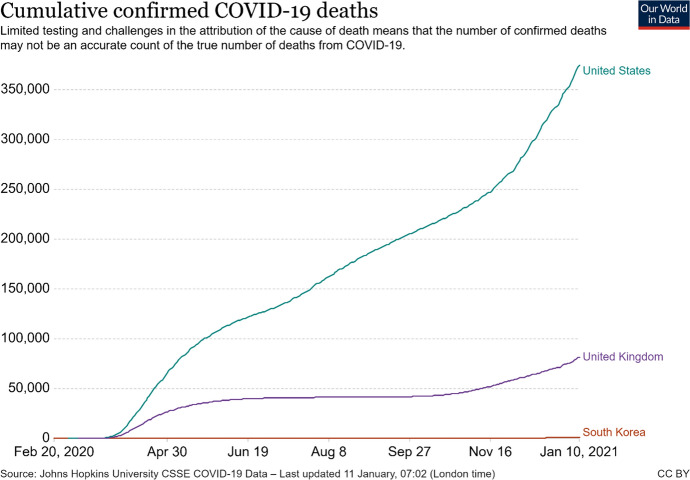
Covid-19 confirmed death in United States, United Kingdom and South Korea. *Source*: https://ourworldindata.org/

The recent pandemic era, caused by the spread of Covid-19, challenged people, economies, and the global health. In order to try to contain and limit the damages caused by the virus, several countries proposed different innovations concerning the testing and screening phase, or the treatment and the prevention to this disease. As a matter of fact, the deaths rate in some countries has been less impactful than in other geographic areas: some of the so called “emerging countries” with high innovative potential (i.e., South Korea) handled the pandemic better than the “advanced countries” (U.S. and United Kingdom), registering less Covid-19 deaths. These data (Fig. 1) have been collected through the platform “Our world in data” (OWID), an online database which includes data on confirmed cases, deaths, and tests performed.

This result is due to different reasons, which might be considered. Certainly, we could not ignore a potential divergence in data gathering; for instance, some countries counted only hospital deaths, whilst others may include deaths in home and the cumulative confirmed Covid-19 deaths could suffer of inaccuracies because of the limited testing and the challenge in attribution of the cause of death. Secondly, even if some of the African and Asian countries do not have better standard of living in terms of income per capita, density of populations or public infrastructures, they already experienced in the past with similar health issues such as Ebola, SARS or Mers. For instance, South Korea learned the lesson of Mers (Han et al., 2020) and was able to perform around 15.000–20.000 detection tests per day, working closely to the private sector: they simply know better how to deal with these plagues. Thirdly, nowadays, emerging countries are developing their own innovative skills and capabilities, building their specific innovations.

In recent times, indeed, emerging countries are becoming the new epicentre of global economic growth and innovation (Shan & Khan, 2016): a part of literature labels the developing economies as the “hotbeds of innovation” (Hadengue et al., 2017; Petrick & Juntiwasarakij, 2011). The innovative process does not depend anymore necessarily on the advanced countries: more often, innovation is born in the emerging economies, and further spreads globally. This phenomenon is well known in the global innovation literature as “Reverse innovation” (RI).

The expression RI, commonly used by the press, but still not investigated in depth in scientific publications (Brem & Wolfram, 2014), was coined by Immelt et al. (2009**)**: it refers to innovations that come from emerging contexts and eventually trickle up in the more advanced ones. Thus,—in antithesis with the Product life cycle Theory of Vernon—we should consider the Reverse innovation as a process, through which innovations internationalize and flow worldwide.

According to several authors, RI is developed with the aim to solve economic, social or health problems (Govindarajan & Euchner, 2012; Govindarajan & Ramamurti, 2011; Lim & Fujimoto, 2019). However, in the literature, innovations related to economic, social, and health issues are commonly defined as Social innovation (SI). This concept has arisen quite recently, and it is enough contested, especially from a definitional point of view. What is new today is that SI, developed in the emerging countries, could have a full potential in the advanced economies too. Indeed, they can improve the life condition of people to health and diagnostics, considering consumers’ purchasing power and people who live at the bottom of the pyramid. The possibility that SI spreads through the process of RI is interesting, because it has many economic and competitive implications for firms in the advanced markets. Nonetheless, these two phenomena have never been linked in previous research and, until today, within the Social innovation literature, scholars focused particularly on the figure of the social entrepreneur/innovator and on the interactions of key actors: it becomes now necessary to study the SI process, and how it could be adequately undertaken.

Therefore, this paper aims at illustrating how Social innovations, reversing their flow, could diffuse from emerging to advanced countries. Further, we want to understand whether Social innovations, once expanded from home to the host market, potentially lose, hold, reduce, or increase their original social connotation.

In line with these goals, in this work we propose a case-study concerning a Social innovation coming from the South Korea, and further spreads in other countries: the “Drive-through” testing center, later become the “Walk-through” screening center, two innovative solutions, which enable monitoring the number of new cases and detect a huge amount of people per day. The initial “Drive-through” testing center, developed in the Province of Gyeonggi, South Korea, was firstly carried out in the United Stated, and later in several countries overseas, such as Italy, Denmark and even in Australia. Simultaneously, in the H Plus Yangji Hospital, this medical device has been turned into the “Walk-through screening center”: within a short time, this enhancement has been introduced in the United States, at the Massachusetts General Hospital (MGH) in Boston. This kind of Social Reverse innovation (SRI) could be potentially decisive in improving the global health situation, reducing the overall impact of the Covid-19.

## Literature review

As suggested by Hadengue et al., Reverse innovation is an “old new idea” (Hadengue et al., 2017, p. 143): new because it addresses a new flow of innovation; old since it could be easily considered as a normal evolution of existing concepts. This dual nature contributes to increasing the uncertainty related to the topic and makes difficult for scholars to give a unique definition of the concept.

Reverse innovation is an under investigated and still nascent phenomenon (von Janda et al., 2018): although it is relatively a new concept, it is attracting academics’ attention. Indeed, Govindarajan and Ramamurti asked themselves why this process occurs now and not earlier. According to the authors, Developed-country Multinational Enterprises (DMNEs) had always the skills and the technologies to develop products for emerging countries, but they did not have enough incentive to do it. With the economic liberalization and the technological changes in the last two decades, things have changed: “DMNEs realized that it is not enough to serve just the premium segment in emerging markets with products that are close variants of those developed for rich countries” (Govindarajan & Ramamurti, 2011, p. 196); instead they have to create products that are specifically developed for their needs.

The first problem scholars face regarding Reverse innovation (RI) is its definition: several authors tried to give an exhaustive explanation. Immelt et al. (2009) introduced this concept for the first time, defining Reverse innovation as products that slide from developed to advanced markets (Immelt et al., 2009); Zeschky et al. (2014) added that a Reverse innovation could be considered as a frugal one that is developed in and for the emerging economies, but that eventually trickles-up to advanced countries (Zeschky et al., 2014). Implementing this trickling up innovation strategy is not as simple as it seems: Reverse innovation is not about creating lower-price products for emerging market, but rather, creating products that answer specifically to these people’s needs (Govindarajan & Euchner, 2012). For this reason, as suggested earlier, we should study this phenomenon as in contrast to the Product Life Cycle Theory (Vernon, 1966): RI should be considered as a process, through which innovations internationalize and flow worldwide.

Von Zedtwitz et al. (2015) try to expand the concept of RI and argue that in the market-introduction based definition, it is quite implicit that is purely the customer perception that determine the reversal character of an innovation: in other words, it is a new product to that specific market, without being new globally. According to the development-based definition “the reversal of innovation denotes a products or service developed in a developing country” (von Zedtwitz et al., 2015, p. 16); thus, the locus of innovation matters for its classification. Subsequently the authors, starting from the four innovation phases—that are ideation, development, primary market, and secondary market introduction (Vernon, 1966)—tried to elaborate the Model of Global Innovation Flows: a binary tree representation with 16 possible global flows of innovation. They define as reverse any global innovation that, at any stage of the process, inverts the flow from a developing to an advanced country; in line with their assumption, while ideation, development and first market represent key moments, secondary market does not. The model came out of the study is built on the distinction between advanced (denoted with “A” in the map) and developing countries (denoted with “D”) and its purpose is to shed light on the RI phenomenon, considering the confusing overlapping with all the other innovation related concepts: i.e. frugal innovation, cost innovation, disruptive innovation etc. (Hadengue et al., 2017). As further finding, the authors define a strong RI as the one “that has at least two of its key innovation phases taking place in a developing country” (von Zedtwitz et al., 2015, p. 18), whereas a weak is an innovation which has merely one of its key innovation phases taking place in a developing country.

As Govindarajan states, “If a multinational won’t do something, a local company will do it” (Govindarajan & Euchner, 2012, p. 16). As the authors underline, the power of a RI consists in being “an instrument for solving some of the world’s most vexing social problems” (Govindarajan & Trimble, 2012a, 2012b, p. 192). Lim et al. (2013) highlight another interesting point of view, stressing the importance of building innovation capabilities for local firms to overcome the “deficiency problem”, i.e. “a deficiency of in-house resources required to generate product innovation in the developing country firm (DCF), and a deficiency of resources of external firms, which could be created from experience in developing an innovative product” (Lim et al., 2013, p. 392).

Lim et al. (2013) believe that creating original products for the unserved lower end market could represent not only a possible solution, but even a sort of competitive advantage over advanced country firms. In accordance with Govindarajan and Ramamurti (2011), business executives should rethink the concept itself of competitive advantage: the EMNEs (acronym for Emerging-country Multinational Enterprises) do not have only “ordinary resources”, instead they possess strengths in low-cost design and manufacturing, efficient distribution and moreover, awareness of local needs. In agreement with Vadera (2020) indeed, “RI as an inevitable process has immense potential to cater to the demands of the 500 million poor population across the world. Companies need to overcome certain challenges like lack of resources, performance, sustainability, Government regulations and lack of infrastructure to take advantage of Reverse innovation saga” (Vadera, 2020, p. 45). Thus, within the global innovation literature about RI, the focus is mainly on the aim of these goods: we should qualify as RI, products developed to satisfy the needs of emerging countries’ customers (Govindarajan & Trimble, 2012a, 2012b; Lim & Fujimoto, 2019).

Innovations, whose primary goal is to suit consumers’ social needs and enable to improve the wellbeing, are well-known within the literature as Social innovation (SI). This concept, as argued by Tracey and Stott (2017) is a contested term, especially from a definitional point of view, with no shared agreed among scholars. On one side, for instance, “it tends to be defined quite generically as the creation and implementation of new solutions to social problems, with the benefits of these solutions shared beyond the confines of the innovators” (Tracey & Stott, 2017, p. 51).

On the other, the burgeoning Social innovation literature, including a large set of organizational forms, has been largely neglected by scholars (Cajaiba-Santana, 2014; Howaldt & Schwarz, 2010; Mulgan et al., 2007a; 2007b), who deepen mostly the social enterprise, which is just one of the manifolds existing types within the Social innovation landscape. Innovation researchers, indeed, outlined greatly the for-profit organizational firm, leaving the SI as a pretty unexplored area, with a referred literature that is “fragmented, disconnected and scattered among different fields”, (Cajaiba-Santana, 2014, p. 42; Van der Have & Rubalcaba, 2016) namely: social entrepreneurship, regional development, social psychology and management. In addition, “there is a lack of literature and research on the Social innovation process” (Lettice & Parekh, 2010, p. 142).

Despite the apparent novelty (Cajaiba-Santana, 2014; Grimm et al., 2013; McGowan & Westley, 2015), SI has mistakenly been analysed as a new phenomenon: according to the authors “[…]it is not: Social innovation as we currently understand it has a rich and fascinating history stretching at least as far back as the cooperative and social business movements of the Victorian era (McGowan & Westley, 2015) and probably much farther, but of course in a general sense Social innovation is as old as civilization itself” (Tracey & Stott, 2017, p. 57).

Based upon another strand of literature, which defines SI as an emerging field (Lettice & Parekh, 2010), the rapid development “has led to a present lack of clarity or overview of what constitutes the field’s own history and current ‘jurisdiction’” (Van der Have & Rubalcaba, 2016, p. 1923).

To compound this issue further, SI has been frequently overlapped with social entrepreneurship, with which has doubtless some analogies, and likewise certain discrepancies (Dawson & Daniel, 2010). As claimed by the authors “Social innovation extends beyond notions of social entrepreneurship in the engagement and ownership of the collective process of developing and steering strategies for social change by the groups involved” (Dawson & Daniel, 2010, p. 18). Indeed, Tracey and Stott (2017) argue that social entrepreneurship, jointly with the social intrapreneurship and extrapreneurship, sets up a different typology of Social innovation.

Concerning the definitional controversy, Social innovations, using Mulgan et al.’s words, are commonly delineated as “innovative activities and services that are motivated by the goal of meeting a social need and that are predominantly developed and diffused through organisations whose primary purposes are social” (Mulgan et al., 2007a, 2007b, p. 8). While broadly accepted, this description may be confusing. More precisely, Social innovations may be differentiated “from business innovations which are generally motivated by profit maximisation” (Mulgan et al., 2007a, 2007b, p. 8); nevertheless, it seems incorrect to affirm that SI can never be driven also by a profit aim. In other words, while social goals represent a focal point, this does not exclude entirely some interest in revenues within these organizations (Altuna et al., 2015). Business and Social innovations are different, although they are overlapping concepts, and for this reason should not be blended together, in order to emphasize SI peculiarities (Pol & Ville, 2009). For instance, both face mostly the same challenges; however, the latter are usually described as more ambiguous and complex (Hall & Vredenburg, 2003), since “social innovators tend to need to satisfy a wider range of stakeholders, with different priorities and potentially conflicting interests and viewpoints” (Lettice & Parekh, 2010, p. 141). Therefore, “these problems are often exacerbated by an increased complexity due to a wider network of diverse stakeholders” (Lettice & Parekh, 2010, p. 155).

In line with the discourse around RI, the role of place in Social innovation, matters: the essence of SI challenges may vary substantially depending on the nature of the institutional context in which social innovators operate. As Tracey and Stott state (2017), this represents a possible area of inquiry for researchers: in the literature, it is now vital to “build theory about how the practice of Social innovation differs, for instance, between countries in the global north versus the global south” (Tracey & Stott, 2017, p. 57).

The future trend foresees an increase of RI both at weak and strong level (von Zedtwitz et al., 2015); besides, concerning their diffusion, social inventions evolve into Social innovations only “when they are widely accepted and used” (Howaldt & Schwarz, 2010, p. 34). So far, it is not yet clear to academics and researchers—but, more important, to business leaders—how to implement this RI process to Social innovations; hence, scholars and researchers should investigate this further.

The key findings concerning the literature review are summarized in Tables 1 and 2.

**Table. 1:** Analytical framework organizing RI key aspects in literature review

Author(s)	Reverse innovation (RI)
*Defenition*	
Immelt et al. (2009)	RI as products that slide from developed to advanced markets
Zeschky et al. (2014)	“RI are cost, good-enough, or frugal innovations that are transferred from the emerging-market environment to developed-country markets”
von Zedtwitz et al. (2015)	“RI as any type of global innovation that, at some stage, is characterized by a reversal of the flow of innovation from a developing to an advanced country, as long as this innovation is eventually introduced to an advanced country’s market”
Hadengue et al. (2017)	“RI is both a new and an old idea”
von Janda et al. (2018)	RI is an under investigated and still nascent phenomenon
Govindarajan and Trimble (2012a, 2012b)	RI as “an instrument for solving some of the world’s most vexing social problems”
*Social connotatoin*	
Govindarajan and Euchner (2012)	“RI is not about hitting low price points; it is about creating fundamentally different products to meet the needs of people in these markets”
Lim and Fujimoto (2019)	RI are products developed in order to satisfy the needs of emerging countries’ customers
Vadera (2020)	“RI as an inevitable process has immense potential to cater to the demands of the 500 million poor population across the world”

**Table 2:** Analytical framework organizing SI key aspects in the literature review

Author(s)	Social innovation (SI)
*Field of study*	
Mulgan, Ali, et al. (2007a, 2007b)	SI literature has been largely neglected by scholars
Cajaiba-Santana (2014)	SI literature is “fragmented, disconnected and scattered among different fields”
Van der Have and Rubalcaba (2016)	The rapid development “has led to a present lack of clarity or overview of what constitutes the field’s own history and current ‘jurisdiction’”
Lettice and Parekh (2010)	SI as an emerging field
Tracey and Stott (2017)	“SI as we currently understand it has a rich and fascinating history stretching at least as far back as the cooperative and social business movements of the Victorian era and probably much farther, but of course in a general sense Social innovation is as old as civilization itself”
*Defenition*	
Mulgan, Ali, et al. (2007a, 2007b)	SI as “innovative activities and services that are motivated by the goal of meeting a social need and that are predominantly developed and diffused through organisations whose primary purposes are social”
Altuna et al. (2015)	Social goals represent a focal point, this does not exclude entirely some interest in revenues within these organizations
Tracey and Stott (2017)	“SI is a contested term. It tends to be defined quite generically as the creation and implementation of new solutions to social problems, with the benefits of these solutions shared beyond the confines of the innovators”
Pol and Ville (2009)	Business and Social innovations are different, although they are overlapping concepts, and for this reason should not be blended in order to emphasize SI peculiarities
Dawson and Daniel (2010)	“SI extends beyond notions of social entrepreneurship in the engagement and ownership of the collective process of developing and steering strategies for social change by the groups involved”

## Methodology

In lines with the research aim, this work proposes a case-study analysis concerning a Social innovation, coming from the South Korea and after implemented elsewhere: the “Drive-through testing center”, subsequently enhanced in the “Walk-through screening center”. The research adopts a case-study methodology, which is useful to investigate a contemporary phenomenon in depth, within its real-life context (Baxter & Jack, 2008; Rosca et al., 2017; Yin, 2003). Moreover, since the boundaries of both the phenomena are not yet clear and evident, this qualitative approach seems to be suitable (Yin, 2003): particularly, the methodology applied is an exploratory single case study, since to the novelty of the topic and also of the specific field of study connected to the new medical devices. We did not select the case randomly: random selection is neither necessary, nor even preferable (Eisenhardt, 1989); conversely, the case has been chosen because of its newness, its relevance and overall, its suitability with the topic. The data employed for the analysis are secondary data i.e., journal articles, websites, and video. All the secondary data were summarized in Table 3, according to the typology. Although secondary data do not represent the best choice, we needed to use them because no other data were available for the Drive-through and the Walk-Through. The adopted protocol provides for an inductive approach: firstly, data were gathered through secondary sources; secondly, we tested the convergence of evidence, verifying the truthfulness of the sources. For instance, we checked where and when the Drive-through and the Walk-through flow, controlling for more than one source.

**Table. 3:** Secondary data sources

*3.a Sources: Journals’ articles*	
Title	Link
BBC “Covid in Europe: How much testing do other countries do?”	https://www.bbc.com/news/54181291
SOCIAL INNOVATION ACADEMY “Social innovation in the times of COVID-19”	http://www.socialinnovationacademy.eu/8-social-innovations-for-covid-19/
CNN “South Korea pioneers coronavirus drive-through testing station” (VIDEO)	https://edition.cnn.com/2020/03/02/asia/coronavirus-drive-through-south-korea-hnk-intl/index.html
Harvard Business Review “Using Reverse innovation to Fight Covid-19”—Ravi Ramamurti	https://hbr.org/2020/06/using-reverse-innovation-to-fight-covid-19
ABC News “What to know about coronavirus testing, including drive-thru clinics, as COVID-19 spreads in US”	https://abcnews.go.com/US/coronavirus-testing-including-drive-clinics-covid-19-spreads/story?id=69584513
ABC News “Doctor details what it's like working at county's first drive-thru coronavirus testing site”	https://abcnews.go.com/Health/doctor-details-working-countys-drive-coronavirus-testing-site/story?id=69685056
National Geographic “How South Korea prevented a coronavirus disaster—and why the battle isn’t over”	https://www.nationalgeographic.com/science/article/how-south-korea-prevented-coronavirus-disaster-why-battle-is-not-over
National Public Radio “South Korea's Drive-Through Testing For Coronavirus Is Fast — And Free”	https://www.npr.org/sections/goatsandsoda/2020/03/13/815441078/south-koreas-drive-through-testing-for-coronavirus-is-fast-and-free?t=1616665873523
THE STRAITS TIME “South Korea dials up coronavirus testing with hospital 'phone booths'”	https://www.straitstimes.com/asia/east-asia/south-korea-dials-up-coronavirus-testing-with-hospital-phone-booths
THE STRAITS TIME “South Korea's 'walk-thru' coronavirus testing booth goes global”	https://www.straitstimes.com/asia/east-asia/south-koreas-walk-thru-coronavirus-testing-booth-goes-global
The Palm Beach Post “County at bottom in virus testing”	https://palmbeachpost-fl.newsmemory.com/?publink=4c1f44fd0_13435aa
HEALTH NEWS FLORIDA “Nonprofit FoundCare To Offer Drive-Through Coronavirus Testing Site In Palm Beach County”	https://health.wusf.usf.edu/health-news-florida/2020-03-15/nonprofit-foundcare-to-offer-drive-through-coronavirus-testing-site-in-palm-beach-county
WRLN “Palm Beach Drive-Thru Testing Site Shuts Down, Overrun By Demand And Short On Supplies”	https://www.wlrn.org/news/2020-03-17/palm-beach-drive-thru-testing-site-shuts-down-overrun-by-demand-and-short-on-supplies
FOUNDCARE “FOUNDCARE SETS UP COUNTY’S FIRST FREE DRIVE-THROUGH TESTING SITE FOR COVID-19 VIRUS”	https://foundcare.org/news/224-foundcare-sets-up-county-s-first-free-drive-through-testing-site-for-covid-19-virus
HEALTH “Drive-Through Coronavirus Testing: How It Works and Which States Offer It”	https://www.health.com/condition/infectious-diseases/coronavirus/drive-through-coronavirus-testing
ANSA.it Lombardia “A Milano drive through per scuole, 500 test al giorno”	https://www.ansa.it/lombardia/notizie/2020/11/13/covid-a-milano-drive-through-per-scuole-500-test-al-giorno_02504734-39d9-4148-b0f5-a711cc418bfe.html
Lombardia Notizie Online “Covid, a Milano il ‘Drive through’ più grande d’Italia”	https://www.lombardianotizie.online/drive-through/
KOREAN BIOMEDICAL REVIEW “H + Yangji Hospital makes accord with Mongolian hospital”	http://www.koreabiomed.com/news/articleView.html?idxno=6194
KOREAN BIOMEDICAL REVIEW “H Plus Yangji Hospital shows off walk-thru system in industry fair”	https://www.koreabiomed.com/news/articleView.html?idxno=9441
KOREAN BIOMEDICAL REVIEW “Korea’s evolving virus tests—from drive-through to walk-through”	https://www.koreabiomed.com/news/articleView.html?idxno=7767
YONHAP NEWS AGENCY “'Drive-through' testing center expand across S. Korea”	https://en.yna.co.kr/view/AEN20200302007000320
AJU BUSINESS DAILY “Walk-thru virus screening system to be patented abroad”	https://www.ajudaily.com/view/20200413172847311#:~:text=The%20walk%2Dthru%20system%20has%20completed%20patent%20applications%20at%20home.&text=The%20glove%20wall%20system%20consists,medical%20staff%20can%20take%20samples
AJU BUSINESS DAILY “Yangi Hospital's 'walk-thru' diagnostic booth wins domestic patent”	https://www.ajudaily.com/view/20200825103723708
THE KOREA HERALD “S. Korea offers coronavirus-related patent information in English”	http://www.koreaherald.com/view.php?ud=20200424000709
Northeastern University’s CENTER for EMERGING MARKETS “REVERSE INNOVATION TO FIGHT COVID-19”	https://damore-mckim.northeastern.edu/reverse-innovation-to-fight-covid-19/
Korea NEWS WIRE “Walk-Thru Developed as the World’s First by H PLUS Yangji Hospital Evolved to Walk-Thru 3.0”	https://www.koreanewswire.co.kr/newsRead.php?no=912262
MASSACHUSETTS GENERAL HOSPITAL “Innovation in Action: New Personal Protective Booths Improve COVID-19 Testing”	https://www.massgeneral.org/news/coronavirus/personal-protective-booths-improve-covid-19-testing

*3.b Sources: Websites*	
Title	Link
FoudnCare Health center	https://www.foundcare.org/
Coronavirus Disease-19, Republic of Korea	http://ncov.mohw.go.kr/en
Korean Intellectual Property Office (KIPO)	https://www.kipo.go.kr/en/MainApp
KIPO Covid-19 Patent Information Navigation	https://www.kipo.go.kr/ncov/sub0702e.html
Korean Intellectual Property Rights Information Service (KIPRIS)	http://engportal.kipris.or.kr/engportal/search/total_search.do
K-HOSPITAL FAIR KOREA Hospital & Medical Equipment Exhibition & Congress	http://en.khospital.org/about/previous_show/

*3.c Sources: Video*	
Title	Link
“'Walk-thru' test booths offer easier access to COVID-19 screening”	https://www.youtube.com/watch?v=uRQ8qNDrxLg&ab_channel=ArirangNews

## The case-study: the Drive-through testing center and its evolution in the walk-through

The Drive-through was inspired by the Drive through at McDonald and Starbuck stores, and it was firstly suggested by the Governor of Gyeonggi Province, Jaemyung Lee. The innovation was developed at the Kyungpook National University Chilgok Hospital in Daegu, in South Korea. This screening center was designed to respond to the increasing number of potential patients in South Korea. The Drive-through should be located away from urban areas, ideally in large parking spaces, whereas it could be implemented also in smaller spot, if well-organized, with an efficient reservation system. The flow of the Drive-through center should be managed with the following phases: entrance, registration, examination, specimen collection instructions and exit. During the entire procedure, the patients must not leave their cars and the healthcare workers (HCW) always must wear the entire personal protective equipment (PPE). Once entered, patients answer to some questions concerning personal information and related symptoms; based on their replies, if Covid-19 is highly suspected, the patients will be transferred to the hospital. At the time of the examination, while the healthcare workers take a nasopharyngeal and oropharyngeal swab through the nearest window, the car ventilation should be kept on the internal circulation mode (Kwon et al., 2020).

The Walk-through is the second evolution of the Drive-through and devised by the Director Kim Sang-il at the H + Yangji Hospital, an example of the highest standard of medical care in all the country, equipped with highly specialized staff and doctors. The Walk-through was born because of two reasons: a large portion of patient visits the screening center on foot and the lack of a large enough parking space to install the Drive-through. Thanks to this testing facilities, the number of tests performed tripled in a day. This testing center, labelled “Safety”, an acronym for “Safe Assessment and Fast Evaluation Technical booths of Yangji hospital”, consists of a plastic booth for one person, with internal negative pressure and rubber gloves inserted. It allows the healthcare staff to test the patient without coming into contact and to reduce drastically the risk of infection (Choi et al., 2020). Safety is also equipped with an interphone that facilitates the communication from the inside to the outside of the cabinet. The flow of Walk-through mostly follows the same principles: entrance, registration, wait, questionnaire, examination, instructions, and exit. Inside the waiting zone, patients could start the registration: social distancing is ensured, since chairs are positioned two meters away. When the patient left Safety, ventilation and disinfection are performed, before the next one enters in the booth (Kim & Lee, 2020).

Both the Drive-through and the Walk-through present of course some limitations and issues: we are going to discuss once at the time. Concerning Drive-through constraint, we could not mention the necessity of wide space to build it in an area with no traffic congestion, the indispensable availability of a personal car, and the possible crowding for unnecessary repeated tests. In this sense, Walk-through has removed the Drive-through insufficiency: it is applicable where patients move on foot and within small space. Although its effectiveness is well recognized, the Walk-through presents some challenges: i.e., the disinfection stage, which is very critical, in terms of disinfectant to use and time of ventilation.

## Discussion

The main obstacle concerning the containment of Covid-19 is related to the difficulties to screen the population without infecting the medical staff. The main point is not only testing people and tracking their movement and contacts, but preserving the functioning of the health system, and the possibility to guarantee treatments to all citizens. In this paper, we present a specific Social Reverse innovation, which enable to get this goal: screening people without endangering the medical staff (Choi et al., 2020).

### The drive-through and its inversion of the flow

**Fig. 2:**
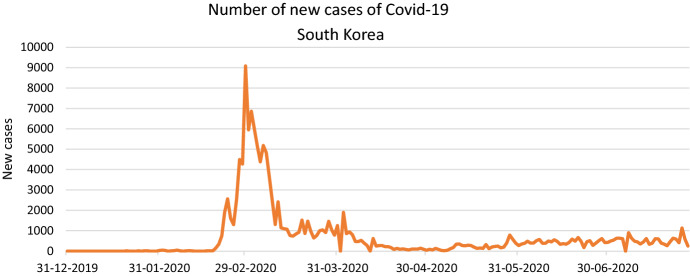
Covid-19 new cases in South Korea. *Source*: https://ourworldindata.org/

The Drive-through was developed in South Korea and opened on the 26^th^ of February, and it represents the first step of the innovation spread and the first evolution of the product: it has not already been released in other countries and there has not yet been its development into the Walk-through. These free Covid-19 screening stations reduce the number of new cases drastically from the beginning of the pandemic. According to the Our World in Data platform (OWID), the number of new cases reached the peak on the 29th of February 2020, with 9.090 new cases and then lowered with average of 700 new cases in the month of July (See Fig. 2). Thus, we could easily affirm that this contraction is an immediate and evident effect of this innovation.

While designed in an emerging country, due to its efficiency in testing and controlling an extensive number of patients, the Drive-through reversed its flow and diffuses in advanced countries: this produces the so called trickling up process, underlined by Immelt et al. (2009). Indeed, the diffusion step of the innovation process involves US and other countries: while the idea of “Drive-through” was not new itself, its potential use against Covid-19 is new. US was the first to adopt and implement the innovation produced in South Korea. Similarly, and subsequently, the Drive-through flows in Mongolia, Italy, Denmark, Australia and doubtless it will be employed in several other geographic areas. For instance, in US the Drive-through was prior carried at the Massachusetts General Hospital (MGH) in Boston, and later all over the States: i.e., at the FoundCare Healthcare Center in Palm Beach County, since the 16^th^ of March, in Colorado, Connecticut and Washington. In Italy, instead, The Drive-through spreads in Milan at the San Paolo and Carlo Hospital, starting from the 16^th^ of November for students and school staff.

Moving from the first evolution of the innovation to the second, the innovators were able to observe and explore the external environment in which the Drive-through works, understanding what elements could represent a possible hurdle to the effective functioning of the screening center.

### The Walk-through and its inversion of the flow

The Walk-through, inaugurated on the 10^th^ of March in South Korea, represents the second evolution of the innovation and—as mentioned before—was designed to solve some of the major Drive-through gaps: Yangji Hospital in the southern Seoul was experimenting the Drive-through with disappointing results. Because of the experience with MERS and SARS, southern-Korean government was aware of the importance of tracking people testing positive for the Covid-19; nonetheless the Drive-through was not succeeding as hoped. Indeed, the hospital location in a crowded neighbourhood and near the subway station, makes it hard, inconvenient, and complex the use of a private car. As the director of the hospital and the inventor Kim Sang-Il stated, he hopes that the Safety booth could act as an efficient example for all the small clinics in the country, who are experiencing troubles in testing people. He applied for a home and overseas patent to protect their intellectual property. Indeed, in April 2020 the H Plus Yangji Hospital entered into an agreement on the public use and development of its intellectual property with the Korean Intellectual Property Office (KIPO). The home patent application has been completed in August 2020. The inventor clarifies that their aim is not to monopolize the innovation; rather he wants to promote a wide use of this innovation, which is completely in agreement with our discourse on the SRI. The office, based on what it states, will provide funds for the commercialization and a brand certification. The Walk-through testing facility will be labelled and commercialized as “K-walk-thru”. The KIPO office, further, stated that the registered patent technology will be unveiled to 61 countries. Starting from April 2020, South Korea will share with the international community its Covid-19 related technologies: indeed, the KIPO has broaden the innovation’s information available on its website, providing especially an English version of the information uploaded. Beside a video tutorial, they share the complete name, the origin of the innovation, and the method. This last section is particularly important for those who are interested in replicating the technology: they indicate the Manufacturer’s name with a phone number and a website; the features and the capacity of the innovation. Based on the Patent act, in a national emergency, it is possible to give up to the patent rights. South Korea has received several requests to share patent’s information and, according to KIPO Commissioner Park Won-joo, their strategy is completely aimed at contributing to the cross-border partnership, to overcome the epidemic crisis. The patent has been also filed with the European Patent Office at the beginning of April 2020. Consequently and because of its effectiveness, hospitals around the South Korea and elsewhere start to endorse their own version of the Walk-through, some independently and others thanks to the help of the inventor and to a relationship of close collaboration and knowledge sharing with the Yangji Hospital. Among the latter, we found the Massachusetts General Hospital in Boston: as the CEO of the American MGH recognized the Walk-through’s high efficacy, he wanted to learn and develop it with the help of the in-house innovation team, the Springboard Studio. After some struggling, and with the precious help of the Korean inventors, MGH was able to install the booths device in all its hospital network in the Boston region, with significant benefits: a more effective sanitization, a strong reduction in the usage of personal protective equipment (PPE), and an increased number of patients tested per day.

Later, the inventors of the Walk-through presented it for the first time at the K-Hospital Fair, held in Seoul on the 21st to 23rd of October 2020. During its presentation, foreign and local visitors expressed great interest.

The inversion of the flow takes place at the diffusion stage, since the health facilities diffuse from the emerging context of South Korea, to the more advanced of US. This reversal is the result of medical agreements and a global collaboration among different institutions. In this advanced network, which involves different and distant geographic areas, knowledge sharing, and cooperation become an imperative. Certainly, without a mutual effort of all the actors embroiled, it is not possible to successfully achieve the inversion of the flow.

### The conceptual framework

**Fig. 3:**
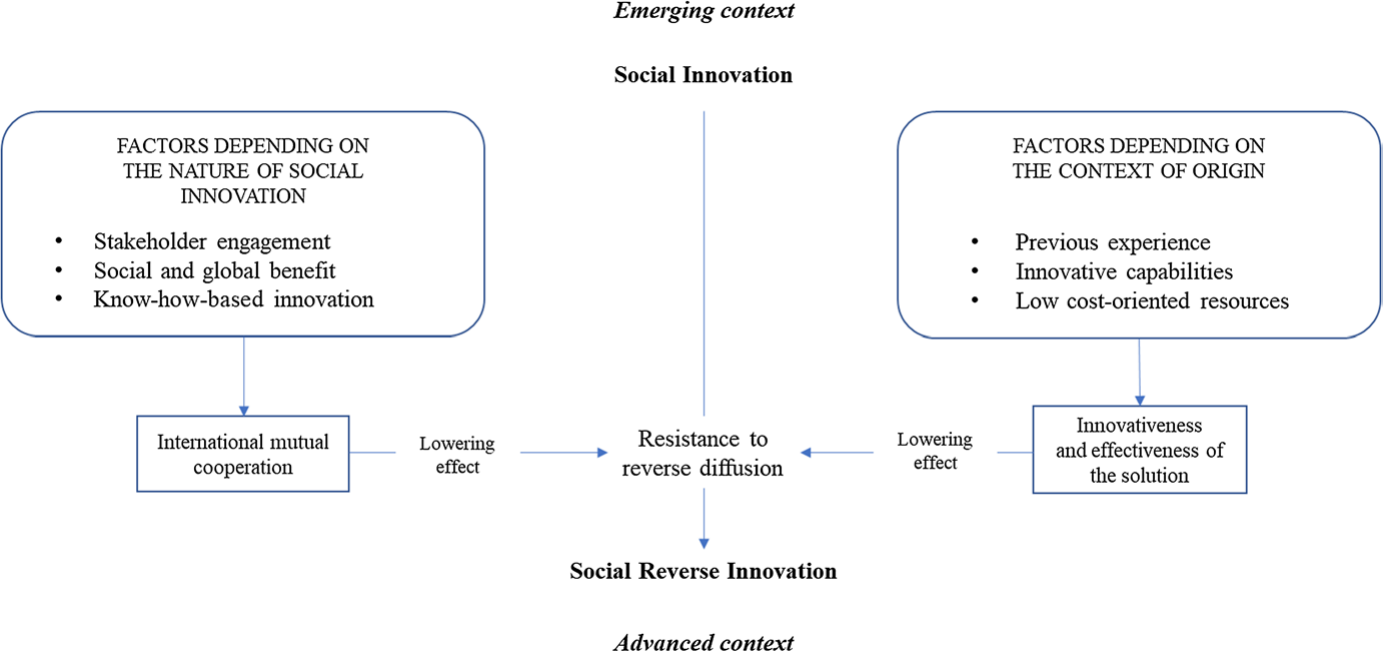
Conceptual framework: How Social innovations spread globally through the process of Reverse innovation

We summarize the key findings of this paper in a conceptual framework that shows the factors affecting the process of RSI (see Fig. 3). In our framework, SI moves from the emerging to the advanced context, thanks to two main types of factors: (a) factors related to context of origin of the SI and (b) factors related to the nature of the innovation. Several elements can be identified among the factors related to SI’s context. Regarding the factors related to the context of origin, the first factor is South Korea's previous experience with similar diseases (Han et al., 2020): as mentioned in the introduction, South Korea learned how to best manage outbreaks such as SARS in 2003 and MERS in 2005. In this way, South Korea developed the know-how to deal with and eventually design effective solutions for the COVID 19 pandemic.

The second factor is the country's innovative capabilities and competencies, similar to other emerging economies (Hadengue et al., 2017) which as Hadengue et al. (2017) argue are becoming the "hotbeds of innovation". As evidence of its innovative capabilities, South Korea has been ranked number one in Bloomberg's Innovation Index for 6 years and ranked second after Germany in 2020. According to the 2020 Global Innovation Index, which is a measure of the innovative capabilities and achievements of the world's economies, South Korea ranks 10th among the 131 economies featured.

The third factor is the resources possessed and available in South Korea and other developing economies to effectively address local needs (Govindarajan & Euchner, 2012) through low-cost solutions in terms of product design, manufacturing, and distribution. Regarding the latter, Drive-through initially used containers as test centres, which were enormously expensive. Later, these were replaced with medical tents to simultaneously save on cost and infection risk (Lee & Lee, 2020).

Together, the three factors described above contributed to strengthening the innovativeness and effectiveness of the solution proposed by South Korea to address the COVID 19 pandemic and thus reducing the potential resistance of advanced countries in adopting a social innovation born in a less developed context. As a result, the reverse flow of the diffusion of this SI occurred without any particular barriers.

Regarding the factors related to the nature of SI, the first factor is stakeholder engagement. The stakeholder involvement typical of each SI is related to the propensity of the actors involved in the innovation to share the knowledge related to the new solution without particular barriers, in order to make the solution more impactful for the intended social problem resolution. In the case analysed in this study, this propensity is demonstrated by the availability of information from the KIPO. Specifically, the agents involved in the process—i.e., the scientists, the physicians, and the inventor in the domestic market—were all characterized by a willingness to distribute knowledge about these medical devices to help solve the pandemic. In fact, when the U.S. CEO of MGH asked for guidance, Director Kim Sang was willing to provide all the needed support. As a result, the stakeholders involved in the development of the Walk-through solution facilitated the diffusion of this SI.

The second factor is the social and global benefit from the innovation developed. Because the pandemic is affecting almost the entire world, South Korea's innovation was easily recognized as something relevant by many countries. As a result, a huge potential global demand for the Walk-through solution emerged around the world making it easier to move the innovation from an emerging to a mature economy (Govindarajan & Euchner, 2012; Govindarajan & Ramamurti, 2011; Lim & Fujimoto, 2019; Vadera, 2020).

The third factor is the nature of SI innovation largely based on know-how rather than physical assets. Because the advanced contexts capable of adopting the new solution (i.e., the United States or Europe) already had the skills and experience to build and produce their own version of the screening center, the only problem in having RI diffusion was the transfer of the relevant knowledge from the emerging context to the advanced context. In fact, the MGH scientists in the Springboard study, once they obtained the know-how from South Korea, were able to build their own screening center.

These three factors, related to the nature of SI, jointly promoted a sense of international mutual cooperation between innovation developers and innovation adopters, which, in turn, lowered the resistance to reversal of the innovation diffusion flow and had a positive impact on the occurrence of SRI.

## Conclusion and limitation

In accordance with Govindarajan and Euchner (2012), while advanced economies were challenged in managing the virus, they were not able to come up with such innovative solutions: indeed, emerging countries go into the game.

The battle against the pandemic Covid-19 is still long, but it is undeniable the central role that these two innovations play within the actual global situation, allowing to screen a major number of people per day, and keeping safe all the healthcare staff involved (Choi et al., 2020).

As discussed before, both the Drive-through and the Walk-through flow from an emerging economy to the more advanced and wealthier, such us the United States and Denmark. This inversion of the flow, scilicet the RI, depending on reciprocal cooperation of the actors involved, is boosted to spread more easily in other countries because of its social power. In other words, in line with what Govindarajan and Trimble (2012a, 2012b) and Govindarajan and Euchner (2012) suggested, the reversal of the flow is more effective since we are dealing with a Social innovation that could be potentially decisive in improving the global health situation, reducing the overall impact of the Covid-19: indeed, early diagnosis is a key factor to prevent the spread of this disease.

The case-study presented not only provides evidence of how Social innovations could diffuse from emerging to advanced countries, by means of the reversed flow; rather, the two innovations prove how SI could hold and increase the social significance, when extended in the host market.

Concerning the methodology, case-studies are considered a powerful instrument when exploring how a phenomenon occurs and studying its progress. Furthermore, among the limitations, a single case-study could present some replicability issues for the analysis: it seems necessary to develop deepen the research and enlarge the analysis, including more case-studies. Gathering data from South Korea was not easy because of the language barrier and privacy. However, the description on the implementation of the process could be enhanced but it suffers for the lack of information concerning the medical devices and both SI and RI literature.

Regarding the limitations, one controversy concerns the definition of South Korea as emerging country: we are discussing about one of the largest economies in the world, with one of the faster growing rates for the last 50 years. However, the index frequently used by investor to classify a geographic area to invest in, disagree about South Korea’s classification. The FTSE Russel (2020) and Woods (2013) categorize South Korea as a developed economy, based on GDP and GNP metrics: South Korea, while having a GDP per capita lower than other developed markets, exceeds the GDP of the emerging.

On the contrary, the MSCI classifies South Korea as emerging: according to the MSCI Market Classification Framework, despite Korea meets the criteria of economic development and liquidity, “it currently fails the accessibility criteria” (MSCI, 2012, p. 1).

Despite the limitations, this paper presents an interesting case-study which gives the possibility to explore the issue connected to social and Reverse innovation, and more specifically on the reverse flow which amplifies the effects of a Social innovation in advanced countries. It also offers an interesting insight into the management of the COVID-19 emergency.

### Future research and managerial implication

The case study presented has different implications: on one side, it highlights that innovation today could origin also from the emerging countries (the so-called Reverse innovation). On the other, if these inverted innovations are Social innovations, the process of diffusion will be enhanced by several factors: the stakeholder engagement, the mutual collaboration of the actors involved, the previous experience and the innovative capabilities of the country where the product is ideated, their resources, in terms of human and economic resources; and finally, the social and global benefit stemming from the innovation. Concerning the latter, a focal point of this work is that these SIs, once launched in the advanced economies, have a full potential to be commercialized also there. Nowadays, indeed, all the innovations with social connotations can be at the same level, independently of the context where they have been created. This, on the one hand, increase the possibilities to innovate. On the other hand, it enhances competition in terms of innovation development. As a result, it is important for scholars to fully explore RI as an internationalization strategy and SI as a type of innovation that can facilitate the diffusion of new solutions from emerging to advanced economies.

Once illustrated that a SI could diffuse from an emerging context to and advanced one, thanks to the inversion of the flow and because of its social connotation, an interesting area for future research emerges. When we are dealing with a SI, is it possible that the reversal of the flow is easier than the traditional process of diffusion? In other words, is it reasonable that a Social innovation developed in an emerging economy spreads to the advanced ones more simply, rather than vice versa? Further, the environmental context of the pandemic, in which these innovations were born and spread, might have significantly affected the flowing from emerging to advanced economies, because of the global state of emergency. Once the virus will be defeated, future research could investigate if our results are confirmed, testing the inversion of the flow under normal conditions.

## Data Availability

All data underlying the results are available as part of the article and no additional source data are required.
